# Hepatitis A outbreak in HIV-infected patients in Southeastern France: questions and responses?

**DOI:** 10.1017/S0950268820000345

**Published:** 2020-04-17

**Authors:** A. Martin, L. Meddeb, J. C. Lagier, P. Colson, A. Menard

**Affiliations:** 1 Aix-Marseille Université, Institut de Recherche pour le Développement IRD, Assistance Publique – Hôpitaux de Marseille (AP-HM), Marseille, France; 2 Microbes, Evolution, Phylogeny and Infection (MEΦI), Marseille, France; 3 Institut Hospitalo-Universitaire (IHU) – Méditerranée Infection, 19-21 boulevard Jean Moulin, 13005 Marseille, France

**Keywords:** Hepatitis A outbreak, HIV, information, men who have sex with men (MSM), vaccination

## Abstract

During the 2017 European hepatitis A (HA) outbreak we assessed HA incidence in our cohort of 2300 HIV-infected patients, implemented preventive measures and evaluated practices and knowledge on sexually transmitted diseases (STD). HA incidence was assessed between 1 January 2017 and 31 December 2017 and included all symptomatic patients with virologically confirmed HA. Preventive measures consisted in identifying at risk and not immunised patients to propose them a free HAV vaccination, and an anonymous survey related to transmission routes of STD and to sexual behaviours. Twenty HA were diagnosed. All were homosexual men recently diagnosed with HIV and another STD. None were vaccinated against hepatitis A virus (HAV). Hospitalisation was required for 52%. We identified 250 patients at risk to acquire HAV and invited them to a free immunisation program. A total of 110 (44%) were vaccinated, of whom 74 responded to our survey. A majority of them (84%) reported recent active anal and oral sexuality with multiple (52%) male partners (81%), and ChemSex consumption (14%). Internet was the meeting link for 58%. Another STD history was found in 69%. One third of these individuals had no idea about STD transmission modes. This HA outbreak pointed the insufficient vaccine coverage against HAV and knowledge on STD, which may be improved by Internet.

## Short report

Hepatitis A virus (HAV) is mostly transmitted in high endemicity countries via the faecal–oral route. In low endemicity countries, HA is most frequently documented after travels abroad, after eating contaminated food or in the setting of secondary transmissions. Notably, HAV has been shown to be transmitted sexually during anal coitus or oral sexual intercourse, and small or even large epidemics have been described among men who have sex with men (MSM) since 1978. In the USA, MSM were identified at higher risk of contracting HAV and transmitting the virus. That's why, the Advisory Committee on Immunization Practices recommended since 1995 their vaccination against HAV. Subsequently, in 2003, the French High Authority of Health (HAS, a national sanitary institution) ended up at the same recommendations. In 2016 and 2017, an HAV outbreak spread in Europe, mostly affecting MSM, either HIV-positive or negative [1]. Our goal was (i) to describe HAV incidence in 2017 in our large cohort of HIV-infected patients followed-up in the infectious diseases units of Marseille university hospital, Southeastern France; (ii) to describe the preventive measures implemented during this outbreak and (iii) to assess among patients vaccinated on this occasion their at risk sexual practices and knowledge on sexually transmitted diseases (STD).

Our cohort included 2300 HIV positive patients followed in three different infectious diseases units of one Marseille University Hospital. The incidence of HAV infection in our cohort of HIV infected patients was assessed between 1 January 2017 and 31 December 2017. We tested for HAV all symptomatic patients and a positive case was confirmed on the basis of concurrent detection in serum of anti-HAV IgM by an immunoenzymatic assay (Architect Abott assays, Abbott Diagnostics, Mannheim, Germany) and HAV RNA by an *in-house* PCR assay [2].

Preventive measures implemented during the HAV outbreak were analysed through the NADIS database. NADIS is a medical file and database designed to follow all HIV positive patients. That database has been used in order to identify at risk patients which mean HIV positive, MSM or bisexual, who weren't immunised against HAV. Then, these patients were contacted by phone or mail to provide a free HAV vaccine immunisation, and in the case they accepted to be vaccinated, to respond to an anonymous questionnaire concerning STD transmission routes and sexual behaviours or practices. This questionnaire was divided into two main parts: the first part was about sexual practices during the 2 previous months, including gender of sexual partners, type of practices (oral sex, anal sex, ChemSex (chemical sexuality)), condom use during oral and anal sex (never, sometimes, always), number of sexual partners (0, 1 regular partner, 2–5, 5–10, 10–30, >30) and places where partners were met (bars/clubs, saunas/hammams, backrooms, internet, street). The second part of the questionnaire was focused on the patients’ knowledge about the risks of STD acquisition: HIV infection; syphilis; hepatitis A, B, C; gonorrhoeae and chlamydiosis. For each of these STD, patients had to select the risks to acquire a given STD by ticking the answers they considered to be correct among food, oral sex, vaginal sex, anal sex, other ways (saliva or urine for example) and none.

Twenty HAV infections were diagnosed in 2017 among our cohort of 2300 HIV-infected patients followed-up in our infectious diseases units for their HIV infection [2]. This represented an overall annual incidence of 0.9%. All these cases involved men between 29 and 52 years of age (mean = 39 years; range: 29–52 years) with homosexual or bisexual practices. These HIV infected patients diagnosed with HAV were younger than HIV infected MSM not immunised to HAV (mean = 39 years; range: 29–52 years *vs.* mean = 45 years; range: 17–71 years, *P* = 0.095). HAV incidence was 3.2% among the 637 MSM or bisexual male patients of our cohort. The 20 patients HAV diagnosed in 2017 had been recently diagnosed with HIV, with a median time of 1.4 years (mean = 1.41 year; range: 0–28 months) since the HIV diagnosis. All of them were under combination antiretroviral therapy (cART). Their plasma HIV RNA load was <35 copies/ml (Veris assays, Beckman-Coulter, USA) for 82% of them, and CD4 cell count was >500/mm^3^ for 60% of them. All these 20 patients had been recently documented with another STD, including syphilis (65%), chlamydiosis (20%), human papillomavirus infection (15%), hepatitis B or C (10%) or gonorrhoeae (10%). Liver cytolysis was observed at time of diagnosis of HAV infection in 30% of the cases, liver transaminases being between 5 and 10-folds the upper normal values, and hospitalisation was required for more than half (52%) of the patients, for a median duration of 4.5 days (Table 1). None of the patients had been vaccinated against HAV. HAV genotype was obtained for 12 of the 20 patients: seven HAV sequences belonged to the VRD-521-2016 ‘UK Travel to Spain’ cluster and five to the RIVM-HAV16-090-Ber:NL ‘NI Europride’ cluster, representing two of the three strains involved in the 2016–2017 European HA outbreak.

**Table 1.** Characteristics of the 20 HAV cases in our HIV + cohort

Age (median (Min–Max) in year)	39 (29–52)	
HIV duration (median (Min–Max) in months)	1,41 (0–28)	
Hospitalisation (median in days)	4.5	
	*n*	%
Males	20	100
Sexual practices		
Homosexual	17	85
Bisexual	3	15
CD4 count		
⩽ 200	1	5
200–500	7	35
>500	12	60
Undetectable viral load	17	82
Antiretroviral therapy	20	100
STD		
Syphilis	13	65
Chlamydiae	4	20
HPV	3	15
Hepatitis	2	10
Gonorrhea	2	10
Cytolysis (between 5 and 10 times the standard)	6	30

Between 1 June 2017 and 30 September 2017 (3 months), we invited patients at high risk to acquire HAV to be vaccinated in our centre in the framework of a free vaccine immunisation program. These patients were among the 637 HIV infected MSM or bisexual male patients in our HIV cohort. In addition, 310 (49%) of these 637 patients were either identified as not immunised against HAV based on a previous HAV IgG serology found negative (*n* = 169; 27%), or were patients for whom we had no result available for a HAV IgG serology (*n* = 141; 22%). Out of these 310 patients, 250 patients (81%) could be contacted, and 110 (44%) accepted to be vaccinated.

In addition, 74 (67%) of the 110 patients who received the vaccine agreed to respond to our anonymous survey. All were men with a median age of 45 years (mean = 45 years; range: 17–71 years). They were HIV positive since a median duration of 7 years (mean = 7 years; range: 0–34 years). All were on cART, with a HIV RNA load <35 copies/ml for 93% of them and a CD4 cell count >500/mm^3^ for 74%. With regard to their sexual behaviour, a majority of the 74 patients (*n* = 62; 84%) reported an active sexuality in the 2 previous months, mostly with male partners (*n* = 60; 81%) or partners of both sex (*n* = 5; 7%). During this period of time, 24 (32%) of the 74 patients had the same sexual partner. The other patients (*n* = 50; 68%) mentioned multiple sexual partners during the 2 previous months, with 2–5 partners for 36%, 5 to 10 partners for 13%, 10 to 30 partners for 10% and >30 partners for 9%. The way for meeting their sexual partner was internet (application on mobile phone and meeting site) for 58% of the patients and dedicated party places for 31% (gay bars in 16%, backrooms in 5%, sauna in 9% and a public park in 9%). With regard to sexual practices during the 2 previous months, anal penetration was reported for 56 patients (76%), with a systematic condom use in 25 cases (34%). A total of 57 patients (77%) had oral sex, protected by a condom in two cases (3%). ChemSex was consumed monthly by two patients (3%), annually by eight (11%), while 21 patients (28%) were unaware of this practice. All 74 patients who responded to the survey had a history of another STD, including syphilis (*n* = 28; 38%), chlamydiosis (*n* = 19; 26%), gonorrhoeae (*n* = 17; 24%) and HBV or HCV infection (*n* = 11; 15%). Nevertheless, 30% of the surveyed patients had no idea of the routes of transmission of STD, including 31 (42%) regarding gonorrhoeae, 29 (39%) regarding chlamydiosis, 23 (31%) regarding HBV/HCV, 15 (20%) regarding syphilis and finally 15 (20%) regarding HAV.

Our infectious disease unit in Marseille, Southern France, did not escape the HAV outbreak that occurred since 2016 in more than 15 European countries including France.

In 2017, the attack rate in our institution was 1% for our whole HIV-infected patients' cohort, and 3% among those patients MSM or bisexual. This is a higher number of cases and incidence than in the other French HIV cohorts from Paris and Lyon for which incidence was reported in 2017. Indeed, 4 and 16 acute HAV cases were reported in these centres, respectively [3, 4]. In addition, about half of the 20 patients HAV diagnosed in our centre were hospitalised, in gastroenterology or infectious disease units. This proportion is similar to the mean proportion observed in previous studies (51%), although it varied considerably, from 21% to 100%, according to the studies. In a previous study, Lee et al. described a prolonged duration of symptoms of hepatitis for HIV positive patients than for those HIV negative ones [5].

The 20 patients who were diagnosed with HAV in our centre were younger than the other HIV-infected patients, and they had been more recently HIV diagnosed (mean = 1.41 year; range: 0–28 months). This suggests the interest to strongly promote HAV vaccination in patients newly diagnosed with HIV in case they are not immunised. Here, none of these 20 cases had been vaccinated against HAV despite recommendations. The HAV vaccine shortage in France that starting 12 months before the 2017 HAV outbreak and that persisted during this outbreak may explain in part that HIV infected patients diagnosed with HAV infection in 2017 in our centre had been recently diagnosed with HIV. In fact, these patients were more likely to miss the opportunity to be vaccinated against HAV following the initiation of the management of their HIV-infection because of this HAV vaccine shortage. At the national scale, it probably contributed to enhance HAV outbreak extent. When the outbreak reached its highest level, we have been able to get HAV vaccines as part of special measures, and vaccinated almost half of the patients recruited among those particularly exposed to HAV. Although the impact on HAV transmission was not measured, vaccinated patients may have reduce the transmission chain as both pre-exposure immunisation as well as immunisation within two weeks post-exposure are effective in reducing HAV infection occurrence. The response to HAV vaccine was likely important as vaccinated patients had high CD4 cell counts (CD4 count >500/mm^3^ for 74%) and a greater CD4 cell count was associated with an increase response to HAV vaccination [6].

The causes and mechanisms of the emergence and drying up of the 2016–2017 HAV outbreak are unknown, as that's the case for many outbreaks. This European HAV outbreak illustrates that such epidemics are linked to one, or few clonal strains. Both genomics variants that we identified to circulate in our geographical area were associated with HAV in MSM in European countries. Thus, RIVM-HAV16-090 was identified among MSM who participated in the EuroPride festival in Amsterdam (23 July to 7 August 2016) and was identical to a Taiwanese strain responsible for 275 notified cases from May 2016, while VRD-521-2016 ‘UK Travel to Spain’ was identified several times among UK MSM returning from Spain. This question if these three strains that circulated in Europe, of which two were detected in our geographical area, might have enhanced contagiousness or potential to escape vaccine stimulated responses compared to other strains. Another reason for the occurrence of the European 2016–2017 HAV outbreak could be related to new sexual practices in the MSM population, but we did not find strong support for such hypothesis neither in the literature nor through our anonymous survey. The implementation of antiretroviral treatment as prevention (TasP) and pre-exposure prophylaxis (PrEP) to prevent from HIV infections might be associated with the phenomena of risk compensation or behavioural disinhibition, which could lead TasP or PrEP users to engage in overall riskier sexual practices and increase their chances of acquiring STD including HA. In Montreal, Canada, an increase rate of STD in PrEP users was observed following the initiation of PrEP [7].

In contrast with previous recent reports on the 2016–2017 HAV outbreak, we investigated among HIV positive MSM patients, unprotected against HAV by prior vaccine immunisation or infection, through a questionnaire, their knowledge about risk factors for HAV infection and about STD transmission. We noticed that these HIV-infected MSM had a very limited knowledge about the route of transmission of major STD, including HA and HIV. Nevertheless they are particularly at risk for these STD, and this is illustrated here by the fact that all had a history of another STD than HAV and HIV. With regard to sexual practices during the 2 months prior the survey, a systematic condom was used by only one third of the patients who reported anal penetration. Nevertheless, one third of them had no idea of the routes of transmission of at least one STD, and of HAV for one fifth of them. The single study conducted in HIV positive MSM explored knowledge about transmission of HIV [8] but no other agents of STD. Here we reported, congruently with Ma *et al*. about HIV transmission [9], a tremendous lack of knowledge about STD among people the more at risk of acquiring such infections in France. This shows the need for improved education and interventions targeting specifically the MSM population. Finally, an interesting finding was that internet was the link for meeting in more than half of the patients who responded to our survey. The network used by million people to meet sexual partners (applications and websites) could be a way to prevent STD [10]. Recently at the peak of HA outbreak, French Authority of Health had a social network communication on Facebook, Hornet or Grindr.

In conclusion, previous findings warrant concurrent real-time surveillance of all sexually-transmitted diseases at local and national scales and point out the dramatically poor knowledge of HIV infected MSM about transmission of HAV and other STD in the new area of ‘condomless sex’. Further studies might determine the efficiency of specific social networks in preventing the spread of STD. Finally, previous data question if the expand in France of TasP and PrEP to control HIV transmission may impact on the transmission of others sexual transmitted diseases, including HA.
